# Management of Super-Refractory Status Epilepticus with Isoflurane and Hypothermia

**DOI:** 10.3389/fneur.2014.00286

**Published:** 2015-01-28

**Authors:** Agzam Zhumadilov, Charles P. Gilman, Dmitriy Viderman

**Affiliations:** ^1^Republican Research Center for Emergency Care, Astana, Kazakhstan; ^2^School of Science and Technology, Nazarbayev University, Astana, Kazakhstan; ^3^School of Medicine, Nazarbayev University, Astana, Kazakhstan

**Keywords:** SRSE, epilepsy, hypothermia, isoflurane, neurotrauma, neurocritical care

## Abstract

Super-refractory status epilepticus (SRSE) is defined as status epilepticus that continues 24 h or more after the onset of anesthesia, and includes those cases in which epilepsy is recurrent upon treatment reduction. We describe the presentation and successful management of a male patient with SRSE using the inhaled anesthetic isoflurane, and mild hypothermia (HT). The potential utility of combined HT and volatile anesthesia is discussed.

## Introduction

A 32-year-old Kazakh male presented with status epilepticus (tonic–clonic seizures) in Almaty, Kazakhstan, where he is employed in a physically active profession. The only noted event before the first seizure was a fever 7 days prior, which subsided without medication. Emergency personnel terminated the initial seizures with intravenous diazepam. Patient refused transport to the hospital but developed a second episode of tonic–clonic seizure several hours later. He was then treated at the hospital’s intensive care unit (ICU) with benzodiazepines and antiepileptic drugs (AEDs), which were unsuccessful. After 7 days of attempted management, the patient was transported by sanitary aviation to the National Medical Holding in Astana, Kazakhstan for treatment by our team.

Upon presentation in Astana, serum glucose and routine serum electrolyte levels were within normal limits. Analyses revealed the following values: hemoglobin, 10.5 g/dL; leukocyte count, 14 × 10^9^ cells/L with neutrophilia; lactate dehydrogenase, 459 U/L; arterial blood gas, pH 7.3, PaCO_2_, 47 mmHg; PaO_2_, 75 mmHg; HCO_3_, 22 mmol/L; and SaO_2_, 87%. Hemoglobin and serum electrolytes indicate normal pulmonary and renal function, while the high LDH may have indicated liver damage, such as what is expected following the recent treatment with benzodiazepines. Both ANA and ENA panels were negative, thereby not supporting an autoimmune dysfunction. CSF was sterile and blood was negative for viruses. ECG showed normal left ventricular function and no regional wall abnormalities.

Head CT initially showed no malformation upon ICU admission in Almaty; however, after 2 days of treatment in Astana, CT showed cerebral edema (compression of third ventricle and flattening of gyri). Edema and ICP were managed with mannitol, and fever with chlortenoxicam. Tumors were not evident from any scan. After 1 week of isoflurane and hypothermia (HT) treatment, head CT indicated a full return to normal morphology.

## Protocols

### Standard protocol

Our ICU uses a standard protocol for evaluation and treatment of status epilepticus. This protocol is as follows:

- management of airway by intubation and mechanical ventilation, which is adjusted to maintain a pulse oximetry of ≥95% and/or PaO_2_ ≥80 mmHg and eucapnia (PCO_2_ of 35–40 mmHg);
- maintaining euvolemia using isotonic fluid to keep central venous pressure equal 8–10 mmHg;
- inotropes to maintain mean arterial pressure above 80 mmHg;
- mild hypothermia (34–35°C);
- maintaining normoglycemia (4–8 mmol/L);
- standard seizure drugs (diazepam, phenytoin, sodium thiopental, propofol);
- antipyretic therapy (paracetamol, ketorolac);
- infusion therapy (normal saline, ringer’s lactate);
- continuous EEG monitoring;
- brain MRI and CT;
- comprehensive toxicology panel including drugs that frequently cause seizures (i.e., isoniazid, tricyclic antidepressants, theophylline, cocaine, sympathomimetics, alcohol, organophosphates, and cyclosporine);
- other laboratory tests: liver function tests, serial troponins, type and hold, coagulation studies, arterial blood gas, AED levels, toxicology screen (urine and blood), and inborn errors of metabolism;
- lumbar puncture if not contraindicated.

### Hypothermia protocol

Hypothermia is induced and maintained using Blanketrol with Maxi-Therm^®^ (Cincinnati Sub-Zero): 34–35°C was achieved within 5 h. Skin, esophageal, and axilla temperatures were monitored hourly during intervention. Rewarming was performed manually over 24 h (~0.12°C/h).

Hypothermia safety monitoring protocol:

- Metabolic status: serum electrolytes were monitored as per clinical routine.
- Respiratory status: blood gases are monitored as per clinical routine.
- Cardiovascular: heart rate, blood pressure, and use of inotropic agents were recorded at baseline and every 4 h, for 96 h.
- Renal status: urine output and body weight were recorded daily during the intervention interval. Serum BUN and creatinine were monitored as per clinical routine.
- Hematological: PT/PTT will be acquired only if bleeding is suspected based upon clinical symptoms or an unexplained fall in hematocrit by more than 10%.
- Complete blood counts are monitored as per clinical routine.
- Infectious disease: results of blood cultures are recorded.

DVT prophylaxis

- If no contraindication, heparin 5000 U subcutaneous, 8 h.

Stress ulcer prophylaxis

- Famotidine 40 mg IVSS ×1.

VAP prophylaxis

- Head of bed to 30°.
- Place in-line closed suction and perform aggressive pulmonary evacuation.

Sedation/analgesia/anti-convulsants

- The use of sedative, hypnotic, and analgesic agents and anti-convulsants is at the discretion of the intensivist.

## Treatment and Outcome

**Figure 1:**
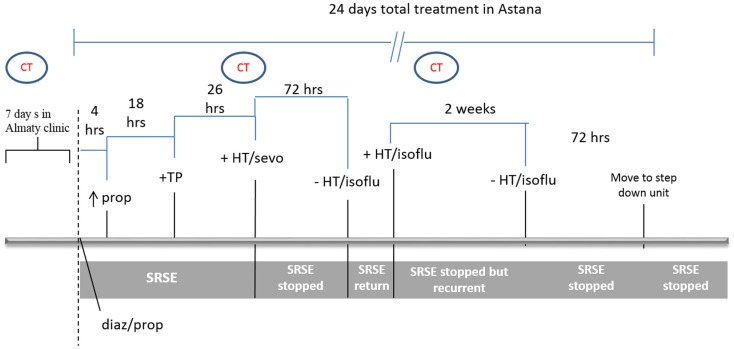
**Clinical course and treatment timeline**. Initial therapy included diazepam (diaz) 0.15 mg/kg, and propofol (prop) 0.027 mg/kg/min. Four hours later, the dose of propofol was increased to 0.09 mg/kg/min. Eighteen hours later, prop was exchanged for sodium thiopental (TP). Twenty-six hours later, general anesthesia was induced with isoflurane (isoflu) and HT initiated. After seizures were dormant for 72 h, the patient was removed from isoflurane and rewarmed, at which point, seizures returned. Isoflurane anesthesia and HT were reinitiated and continued for 2 weeks, during which the patient was stable without super-refractory status epilepticus (SRSE). Attempts to withdraw from treatment were unsuccessful over these 2 weeks. The patient was then gradually removed from isoflurane and HT, remained seizure-free for 72 h and was then released from the ICU.

Figure 1 shows a schematic of the treatment/response timeline. Initial therapy consisted of diazepam 0.15 mg/kg (10 mg bolus; this dose was repeated after 5 min) and propofol 0.027 mg/kg/min, valproic acid 2 g/day, and carbamazepine 200 mg twice daily. This therapy was not sufficient to reduce seizures after 4 h and the dose of propofol was increased to 0.09 mg/kg/min. To reduce the continued hepatic damage (indicated by treatment and LDH levels), propofol is a good first choice (1); however, it failed to alleviate epileptic activity. As the combination of these drugs continued to fail at 22 h, propofol was exchanged for sodium thiopental (0.04 mg/kg/min) and 16 h later was increased to 0.07 mg/kg/min. Despite this therapy seizures continued (monitored by continuous EEG recording), resulting in hemodynamic instability with profound hypotension demanding vasopressors (epinephrine, 0.1 μg/kg/min and phenylephrine, 0.09 μg/kg/min) with continuous normal saline infusion.

After the first 48 h of attempted treatment, we then initiated general anesthesia with isoflurane [minimal alveolar concentration (MAC) 1.0] and HT (34–35°C). Isoflurane was associated with ameliorated epileptic discharges with adequate, sustained EEG burst suppression within minutes of initiating. Isoflurane was partly chosen because of its lesser association with drug-induced hepatitis (2). Isoflurane MAC was then decreased to 0.7 and was not associated with worsened hemodynamic instability. The administration of sodium thiopental and diazepam was stopped and, 30 min after discontinuing these drugs, hemodynamics started to stabilize. Epinephrine and phenylephrine were decreased to 0.075 and 0.05 μg/kg/min, respectively. Termination of seizures was determined by EEG and no seizure activity was registered during the immediate 72 h after beginning isoflurane administration. The patient was then rewarmed and isoflurane inhalation was gradually stopped but, despite the continued administration of valproic acid and carbamazepine, seizures reoccurred. Isoflurane anesthesia (MAC 0.7) was again initiated and the patient was recooled to 34–35°C. This dosage was considered minimal to control seizures. Seizure control efficacy was monitored by continuous EEG.

Two weeks later, isoflurane was gradually decreased to 0.2 MAC over 24 h and finally stopped. The patient was rewarmed and convulsions did not reoccur. Several unsuccessful attempts had been made to remove the patient from HT and isoflurane over the 2-week interval. Standard anticonvulsant therapy (valproic acid and carbamazepine) was continued and 72 h later the patient was discharged from the ICU to the step down unit and a week later from the hospital.

In total, the patient was mechanically ventilated for 24 days and this was complicated by ventilator-associated pneumonia. He was successfully extubated and started on an intensive rehabilitation program with rapid improvement. After discharge to his home, brain magnetic resonance imaging did not show any signs of pathology 3 weeks after being extubated. A cognitive function assessment was not performed by a psychologist at that time since his mental and psychological functions were significantly recovered. He returned back to work at 2 months after the event with excellent and complete functional recovery and no known neurological or psychological deficits.

## Background

Refractory status epilepticus (RSE) is continued seizures after the failure of two or more AEDs. Common intravenous agents such as diazepam, lorazepam, midazolam, and phenytoin used to treat RSE have frequent problems with toxicity or efficacy (3–5). SRSE is defined as status epilepticus that continues 24 h or more after the onset of anesthesia, including those cases in which the status epilepticus recurs on the reduction or withdrawal of anesthesia. Status epilepticus is a neurological emergency that requires immediate recognition and treatment and carries a significant economic impact on the healthcare system (6). Annual incidence in population-based studies ranges from 10 to 60 cases per 100,000 persons, annually (7, 8).

Super-refractory status epilepticus therapies and their outcomes were recently and thoroughly summarized (9): 1168 SRSE patients were described to have received 19 possible treatments and all patients received some treatment combination. Midazolam was used most frequently in 50% of all SRSE patients. Propofol and thiopental/phenobarbital were also common and used in 12 and 16% of cases, respectively. Overall, the expected outcomes for SRSE are poor; only ~33% of patients recovered to baseline function. The authors note that the source of the SRSE inducing-injury is likely a dominant determinant of ultimate outcome.

While no comprehensive trial has been performed with isoflurane for SRSE treatment, anecdotal evidence and case studies suggest that it is an efficacious alternative when standard drugs fail or are contraindicated (such as with some concomitant chemotherapy). There may, however, be limits to its utility as prolonged use in two patients (34 and 87 days) was associated with subcortical white matter hyperintensities on MRI, which was reversed upon discontinuation (10). Short-term treatment appears to associate with manageable risk, and in the small number of reported cases, isoflurane was able to ablate seizures within minutes of anesthetization (11). Ferlisi and Shorvon reviewed the data from 27 refractory epileptic patients that were treated with inhalational anesthetics (7 independent publications including those cited here) and conclude that, indeed, this strategy initially controlled seizures in 100% of cases (9). However, there is an elevated risk for adverse events, leading one to conclude that isoflurane’s utility is best reserved until other treatments have failed (which is generally the condition for SRSE).

The 2012 Guidelines for the evaluation and management of status epilepticus (12) provides a brief assessment for alternative epilepsy treatment including both inhalational anesthetics and HT. Hypothermia is attractive for several reasons: reduced edema, fever prevention, strong acute efficacy for blocking seizures, and a relatively low risk for adverse effect (13). Unfortunately, HT also tends be a temporary solution, as epilepsy often reoccurs upon warming. Investigations of HT for treating cardiac arrest indicate that the method of inducing HT, the stability of body temperature control, duration of HT and rate of rewarming all impact its efficacy, and differences in the these variables between centers are important to consider to understand inconsistencies between outcomes following a neurological injury (14, 15). For example, in one meta-analysis of traumatic brain injury, the authors included studies that used many different techniques including different methods for measuring core body temperature, different temperature ranges over the HT maintenance phase, and different rates of rewarming (16). Some degree of HT can be easily and crudely implemented in clinical units and therefore there is an enormous degree of variability between study techniques (17). These questions need to be refined and answered to provide the best treatments to SRSE.

## Discussion

In this case, standard therapy for status epilepticus was not effective; however, inhaled anesthesia with isoflurane paired with HT showed excellent efficacy and was without significant adverse effect (mild hypotension was managed with normal saline infusion and low dose norepinephrine). Since SRSE incidence is too low to readily perform a properly designed study, we intend for this case study to serve as an illustration of the potentially beneficial role of inhalation anesthesia with isoflurane and mild HT in SRSE management.

Inhalational anesthesia with isoflurane and HT were effective in SRSE management, when even multiple anticonvulsive drug therapies were ineffective. This treatment was also better tolerated compared to high dose thiopental and other anticonvulsive medications. Persistent hypotension during the first days was well corrected with normal saline infusion and low dose vasopressors. Treatment with high dose diazepam and thiopental was not able to fully terminate convulsive activity, caused more profound hypotension and elevated hepatic enzymes (ALT, AST). Electrolyte imbalances, kidney or liver damage, and diabetes were excluded according to normal arterial blood values. During the first 24 h of isoflurane anesthesia, sodium thiopental was gradually decreased and finally stopped. The patient was given isoflurane anesthesia with MAC 0.7 and AEDs (carbamazepine, phenytoin, phenobarbital at standard doses). During 20 days of ICU treatment, several attempts to stop isoflurane anesthesia were made but seizures returned. After 24 days in treatment (17 days with isoflurane and HT), the patient was able to be taken off of anesthesia and recovered rapidly, returning to work within 2 months.

General anesthesia is effective for the largest number of patients with status epilepticus that are refractory to conventional anticonvulsant therapy. However, compared to other general anesthesias, isoflurane has minimal reported organ toxicities, is rapidly titratable, and moreover, isoflurane lowers the shivering threshold (18), thereby functioning well as a partner for HT. The application of isoflurane here was not associated with a severe adverse event. This is not consistent with previous reports on the adverse effects of inhalational anesthesia for RSE patients. We believe that the seizure-causing injury itself may be a significant determinant of serious adverse events during treatment and this is highly heterogeneous for epilepsy patients. It would be useful to determine the associations between the source of SRSE and the efficacy/risk of treatments; however, this may require a sample size far exceeding the expected presentation frequency.

There is a general consensus that isoflurane is associated with more adverse effects; however, we are not aware of a retrospective analysis that permits the dissociation of the adverse events associated with isoflurane versus the injury itself or other treatments that always overlap with isoflurane therapy (11). Compared to established epilepsy treatments (benzodiazepine plus AED), there is not sufficient evidence that isoflurane is on average an improved treatment for status epilepticus, however, by its nature, SRSE is a failure of the established treatments. There is no best known treatment for SRSE largely because it is a rare complication. We propose that, despite the small number of reported studies, isoflurane and HT may be a reasonable treatment after standard treatments have failed (e.g., in SRSE).

Hypothermia is a therapy that deserves further study for SRSE because of the extensive preclinical experimental evidence of neuroprotection for epilepsy and almost immediate seizure-terminating properties in over 80% of patients (19). However, HT possesses significant potential complications and side effects, including acid–base imbalance, infection, and coagulopathy. The level of HT to be effective is also uncertain, and it has been suggested that only HT between 30 and 34°C has an appropriate risk-to-benefit ratio (20). The shifting standards of the use of therapeutic HT for cardiac arrest have illuminated the importance of prophylactic antipyresis but are associated with convincing evidence that the utility of HT may be more limited than recently believed (21). We need to ask the same question for status epilepticus; stated crudely – is it safe and does it work (does it have a favorable risk-to-benefit ratio)? The safety profile of controlled mild HT is considered manageable and, for RSE, eliminating seizure activity quickly and in 100% of almost all patients (even if temporarily) is a truly significant benefit. On a case by case basis, by the definition of SRSE, if isoflurane or HT stops seizure activity, then it is superior to the failed treatment (in that specific case).

## Concluding Remarks

If even short epileptic episodes cause excitotoxicity and brain damage, then it is imperative that reliable secondary strategies be adopted for when AEDs fail. While the utility of inhalational anesthesia and HT in neurocritical care is debatable, it is clear that these therapies can be an effective treatment for intractable seizures in SRSE. At the very least, temporary amelioration of epileptic activity is expected to improve the long-term outcomes by limiting the excitotoxic injury.

## Conflict of Interest Statement

The authors declare that the research was conducted in the absence of any commercial or financial relationships that could be construed as a potential conflict of interest.
